# Silver-Copper Oxide Heteronanostructures for the Plasmonic-Enhanced Photocatalytic Oxidation of N-Hexane in the Visible-NIR Range

**DOI:** 10.3390/ma12233858

**Published:** 2019-11-22

**Authors:** Hugo Suarez, Adrian Ramirez, Carlos J. Bueno-Alejo, Jose L. Hueso

**Affiliations:** 1 Institute of Nanoscience of Aragon (INA) and Department of Chemical and Environmental Engineering, C/Poeta Mariano Esquillor, s/n; Campus Rio Ebro, Edificio I+D, 50018 Zaragoza, Spain; 2 KAUST Catalysis Center (KCC), King Abdullah University of Science and Technology (KAUST), 23955 Thuwal, Saudi Arabia; 3 Networking Research Center on Bioengineering, Biomaterials and Nanomedicine (CIBER-BBN), 28029 Madrid, Spain; 4 Instituto de Ciencia de Materiales de Aragon (ICMA), Consejo Superior de Investigaciones Cientificas (CSIC-University of Zaragoza), 50018 Zaragoza, Spain

**Keywords:** plasmonic photocatalysis, silver-copper oxide, VOCs remediation, full-spectrum photoresponse

## Abstract

Volatile organic compounds (VOCs) are recognized as hazardous contributors to air pollution, precursors of multiple secondary byproducts, troposphere aerosols, and recognized contributors to respiratory and cancer-related issues in highly populated areas. Moreover, VOCs present in indoor environments represent a challenging issue that need to be addressed due to its increasing presence in nowadays society. Catalytic oxidation by noble metals represents the most effective but costly solution. The use of photocatalytic oxidation has become one of the most explored alternatives given the green and sustainable advantages of using solar light or low-consumption light emitting devices. Herein, we have tried to address the shortcomings of the most studied photocatalytic systems based on titania (TiO_2_) with limited response in the UV-range or alternatively the high recombination rates detected in other transition metal-based oxide systems. We have developed a silver-copper oxide heteronanostructure able to combine the plasmonic-enhanced properties of Ag nanostructures with the visible-light driven photoresponse of CuO nanoarchitectures. The entangled Ag-CuO heteronanostructure exhibits a broad absorption towards the visible-near infrared (NIR) range and achieves total photo-oxidation of n-hexane under irradiation with different light-emitting diodes (LEDs) specific wavelengths at temperatures below 180 °C and outperforming its thermal catalytic response or its silver-free CuO illuminated counterpart.

## 1. Introduction

Global warming, massive deforestation for urbanization and increasing contamination caused by mankind practices are contributing to the alarming rise of pollutant emission levels worldwide. Among these contaminants, the exposure to volatile organic compounds (hereafter VOCs) is recognized as a serious hazard to human health contributing to skin, respiratory, and cancer diseases [1,2,3]. Even if the exposure dose is very low, it has become an issue of increasing interest since many of the VOCs emitting sources are not only stemming from big factories or production plants. Indoor sources such as tobacco smoke, solvents, paints, furniture, computer, personal use products, etc. are continuously contributing to the VOCs emissions in indoor habitats [1,2,3,4,5]. There are currently different exploring technologies devoted to VOCs remediation including the use of plasma discharges [6,7,8,9], microwaves combining absorption–desorption–combustion steps [10,11,12], photodegradation [2,5,13,14,15,16,17,18,19,20], and adsorption/catalytic oxidation [2,21,22,23,24,25,26,27,28,29]. Total oxidation of VOCs promoted by conventional catalysts represents one of the most appealing alternatives. Noble metals are able to completely oxidize VOCs into CO_2_ and H_2_O at mild reaction temperatures [22,23,25,30,31,32,33,34,35,36,37,38,39]. Transition metal oxides and complex metal oxides (i.e., rare earth element-based perovskites) are also excellent VOCs oxidation candidates that operate at relatively mild temperatures without incurring in the burdening costs of noble metals [3,8,16,37,38,39,40,41,42,43,44,45]. Alternatively, the use of inexpensive arrays of photocatalysts based on titania (TiO_2_) has become one of the most important research fields towards the sustainable remediation of VOCs [5,20,46,47,48,49,50,51]. The advantages of using solar light or low consume artificial lights to promote VOCs oxidation at room temperature is being actively pursued. Current limitations are found either in the weak response of the most active semiconductor photocatalysts (i.e., TiO_2_, ZnO) beyond the UV range (that only represents 4%–5% of the full solar spectrum) or in the rapid electron-hole recombination rates detected in transition metal oxide semiconducting photocatalysts with expanded absorption capacities towards the visible-near infrared (NIR) ranges (i.e., MO_x_, M = Cu, Fe, Mn, Co) [2,43,52,53,54,55,56,57,58].

To overcome these drawbacks, current research interests in VOCs remediation are focused on the development of hybrid nanomaterials combining metal oxides, metal transition oxides and/or noble metals with photocatalytic response expanded towards the visible-NIR ranges [16,21,59,60,61,62,63,64,65,66,67,68,69]. Metallic nanoparticles can play a determining role in expanding the absorption range of regular metal oxides such as titania (TiO_2_) and/or reducing the electron-hole recombination rates of metal transition oxides (i.e., MO_x_ (M = Fe, Mn, Co, Cu) [2,3,19,58,59,70,71,72,73,74,75,76,77,78,79,80,81,82,83,84,85,86,87,88,89,90]. Furthermore, metallic nanoparticles have become particularly relevant due to their plasmonic properties [2,76,81,86,91,92,93,94,95,96]. The localized surface plasmon resonance (LSPR) is a unique characteristic of these materials (normally Au, Ag, Pt, or other noble metals), which can extend the absorption of light towards the visible light spectrum [69,87,88,89,90]. Thus, LSPR greatly supports the utilization of the solar spectrum [69,81,92,97,98,99,100,101,102,103,104]. In addition, plasmonic nanoparticles may play active roles as sensitizers (via antenna effects) or accommodate charges from semiconductors upon forming effective Schottky metal-semiconductor junctions as in well-established noble metal-TiO_2_ hybrid systems [69,92,97,98,99,100].

In the present work, we aimed at exploring the synthesis of a hybrid heterostructured catalyst combining the plasmonic properties of Ag and the p-type semiconductor capabilities of CuO [3,101,102,103,104]. Both materials are abundant, affordable, and exhibit a strong potential for full-range photocatalytic applications. Previous studies based on plasmonic silver nanostructures [76,92], Cu_x_O_y_ systems [105,106,107] or in the combination of silver-copper alloys [108,109,110,111,112], silver-copper oxide heterostructures [109,113,114,115] or even silver-copper oxide decorating TiO_2_ [70,108] have already proved their potential not only in visible-NIR expanded photocatalysis [70,114,116], but also in solar harvesting, electrocatalysis, bacteria disinfection, field emission enhancement or the formation of novel superconducting structures [55,108,109,111,114,115,116,117,118,119,120]. Herein, we have demonstrated that these Ag-CuO heterostructures with an intertwined configuration maximize the plasmon-semiconductor interaction. As a result, a very active hybrid heterostructure with full-spectrum LED-driven photoresponse towards the total oxidation of n-hexane has been developed. Its photocatalytic response becomes especially photoactive upon irradiation with LED wavelengths of 460 nm. The heterostructure fully photooxidizes n-hexane at temperatures below 180 °C and outperforms its silver-free CuO counterpart thereby outlining the relevance of the silver nanostructures entangled with the CuO nanotubes in the Ag-CuO hybrid. To the best of our knowledge, this study presents the first example of full-spectrum photocatalytic assisted VOCs oxidation in a diluted gas phase with this kind of Ag-CuO configuration.

## 2. Materials and Methods

### 2.1. Synthesis of the Photocatalysts

The Ag-CuO heteronanostructures were synthesized following a protocol reported elsewhere [121]. Cu(NO_3_)_2_·3H_2_O (3.2 mmol, Aldrich, Saint Louis, MO, USA, 99.9%) and AgNO_3_ (3.1 mmol, Aldrich, Saint Louis, MO, USA, 99.9%) were dissolved in 3 mL of deionized water. The resulting silver-copper solution was added to an aqueous solution of NaOH 3 M (4 mL, Aldrich) with vigorous stirring for 6 h under an inert Ar atmosphere. The resulting black adduct that was vacuum filtered, washed with water, and dried at 100 °C for 2 h. The solid was calcined at 350 °C for 6 h. In order to obtain silver-free CuO nanostructures, an analogous synthesis protocol was followed but skipping the addition of the silver salt precursor. The synthesis of the photocatalysts has been performed at the platform of Production of Biomaterials and Nanoparticles of the NANBIOSIS ICTS, Spain, more specifically by the Nanoparticle Synthesis Unit of the CIBER in BioEngineering, Biomaterials & Nanomedicine (CIBER-BBN).

### 2.2. Characterization Techniques

Transmission electron microscopy (TEM) analysis was carried out using a T20–FEI microscope (Hillsboro, OR, USA). Aberration corrected scanning transmission electron microscopy (STEM) images were acquired using a high angle annular dark field detector in a FEI XFEG TITAN microscope (Hillsboro, OR, USA) at 300 kV equipped with a CETCOR Cs-probe corrector. High-resolution transmission electron microscopy (HR-TEM) images were acquired with the aid of a FEI TITAN^3^ electron microscope operated at 200 kV. Elemental analysis was carried out with Energy Dispersive Spectroscopy (EDS) (EDAX, Mahwah, NJ, USA) detector using single point and scanning profiles. The samples were drop-casted onto Ni mesh grids. The N_2_ adsorption/desorption analyses were performed with the aid of a Micromeritics ASAP2020 analyzer (Norcross, GA, USA). 80–100 mg of the catalyst was degassed at 90 °C for 12 h. The surface area was determined using the Brunauer–Emmett–Teller (BET) method rendering a value of 4.7 m^2^·g^−1^ for the Ag-CuO tubular heterostructures. Scanning electron microscopy (SEM) analysis was carried out with FEI-Inspect S50 equipment (Hillsboro, OR, USA). X-ray diffraction patterns were obtained in a PANalytical Empyrean equipment (Malvern, UK) in Bragg Brentano configuration using Cu-K radiation and equipped with a PIXcel1D detector. The absorption spectra were acquired with a JASCO V-670 UV-VIS-NIR spectrophotometer (Tokyo, Japan) and the aid of an integrated sphere accessory.

### 2.3. Photocatalytic Reaction Setup

The experimental setup designed to carry out the photocatalytic degradation of n-hexane has been previously described elsewhere [20,122]. Briefly, the reaction was conducted in a home-made system comprising a quartz cell (50 × 10 × 5 mm^3^ (height × width × length). The cell was illuminated by two high power LEDs (LedEngin) cooled with the aid of custom-designed fans. Different LEDs wavelengths were individually tested. Each LED operated with different power: 405 nm (3.9 W), 460 nm (2.2 W), and 940 nm (3.2 W). Selected light irradiances ranged from 7 to 11,000 mW/cm^2^ (based on LED specifications and experimental setup) and LED powers were programmed with the aid of an external power supply unit (ISO-TECH, IPS-405, 0–40 V). The temperature of the catalytic bed containing 300 mg of Ag-CuO during irradiation was monitored with the aid of a K-type thermocouple. The photocatalytic experiments were performed with a total flow of 50 mL·min^−1^ of gas containing 200 ppm of n-hexane (space velocity = 10,000 h^−1^). Conventional heating experiments were carried out with a home-built system consisting of an aluminium holder designed to heat the same reactor area as in the quartz reactor used for the photocatalytic test [20]. The inlet final concentration of n-hexane was achieved upon mixing with the proper flow rates, n-hexane in N_2_, O_2_, and synthetic air (all purchased from PRAXAIR España S.L.U., Madrid, Spain), in order to get the different total flow rates assessed. After an equilibration period of 30 min that served us to evaluate the adsorption of n-hexane in the dark, the LED lights were turned on for different time intervals and the gas effluent outlet analyzed by gas chromatography (Agilent 3000 Micro GC, Santa Clara, CA, USA). An OV-1 and a PPQ column in line with a thermal conductivity detector (TCD) were employed to separate and detect the different gas compounds. The steady state final concentration achieved was always ≤10 ppm of n-hexane when maximum LED power was used. This steady state was always achieved within minutes regardless of the experimental settings. Under the conditions used, the n-hexane detection limit was 3 ppm and CO_2_ was the only oxidation product detected. Maximum error in the mass balance closures for carbon and oxygen in this work was ±2%.

## 3. Results

### 3.1. Characterization of the Silver-Copper Oxide Plasmonic Photocatalyst

**Figure 1:**
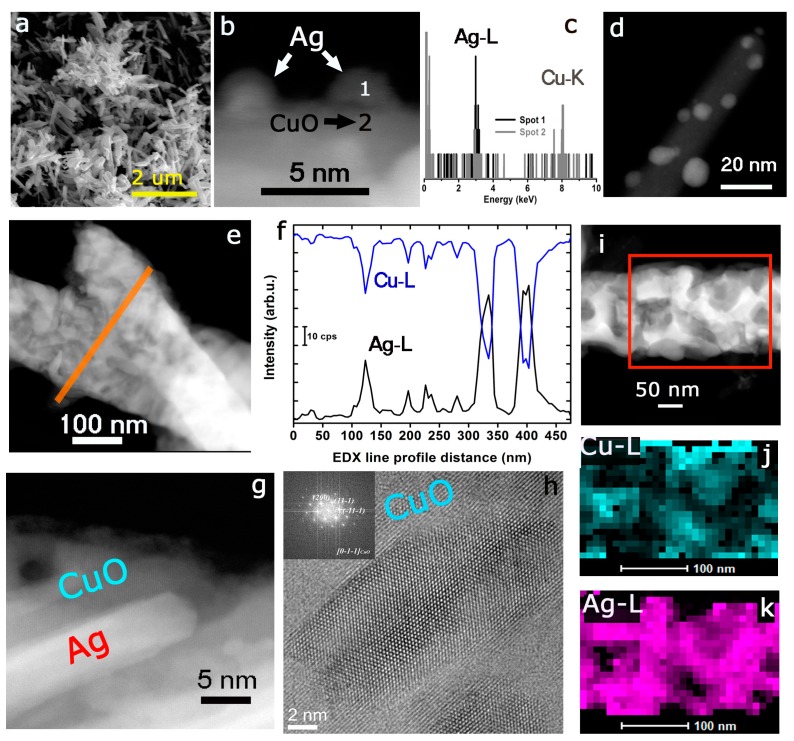
Morpho-chemical characterization of the silver-copper oxide photocatalyst: (**a**) SEM representative image accounting for the tubular shape of the Ag-CuO hybrids; (**b**) High Angle Annular Dark-Field (HAADF)-STEM image of small Ag nanoparticles (NPs) in the outer area of the nanotubes dispersed in a Cu-based matrix, the numbers refer to specific areas for EDX spectra acquisition; (**c**) EDX analysis of selected spots in (**b**) accounting for the specific present of Ag or Cu; (**d**) HAADF-STEM image of a CuO nanotube with Ag NPs decorating in the external region; (**e**) STEM image of individual nanotubes and EDX line profile analysis performed and plotted in (**f**); (**f**) evolution of Ag-L and Cu-L intensities across the EDX line profile analysis depicted in (**e**); (**g**) HAADF-STEM image accounting for the presence of anisotropic Ag shapes embedded within the Cu-based matrix; (**h**) HR-TEM image corresponding to the Cu-enriched region accounting for the presence of a monoclinic CuO phase (inset: Fast Fourier Transform (FFT) image with indexed CuO planes in the [0-1-1] direction); (**i**) HAADF-STEM image of a fraction of Ag-CuO nanotube containing bigger aggregates (the square accounts for the selected area for EDX mapping analysis); (**j**) EDX map accounting for the Cu-L edge (wt %) intensity in the selected area of (**i**); (**k**) EDX map accounting for the distribution of the Ag-L edge intensity (wt %) in the selected area of (**i**).

The morphological evaluation of the Ag-CuO heterostructures by SEM revealed the presence of tubular-shaped structures (Figure 1a) [121,123,124]. A more detailed analysis by HAADF-STEM in combination with EDX analysis confirmed the presence of both Ag and Cu species as segregated elements. Figure 1b–d reveal the corresponding analysis of the outer surfaces of the tubular structures. Small Ag nanoparticles are supported onto the Cu-based surface (Figure 1b,d). An extended EDX line profile analysis across two individual nanotubes further confirmed the alternating presence of either silver or copper elements (see Figure 1e–f).

It is also worth mentioning that Ag was identified with different morphologies, including small segregated nanoparticles, rod-shaped anisotropic structures (Figure 1g), and non-uniform aggregates dispersed along the tubular-shaped CuO matrixes (Figure 1i). HR-TEM analysis of the Cu-based regions confirmed the presence of a well-defined orientation corresponding to a CuO crystalline phase (Figure 1h). The FFT inset in Figure 1g corresponded to the orientation of the CuO fraction in the [0-1-1] direction. The (200), (11-1), and (-11-1) planes were identified and matched with a C2/c monoclinic system. EDX mapping of the intensities of Ag-L and Cu-L signals further assessed the entangled distribution of Cu (Figure 1j) and Ag phases (Figure 1k).

**Figure 2:**
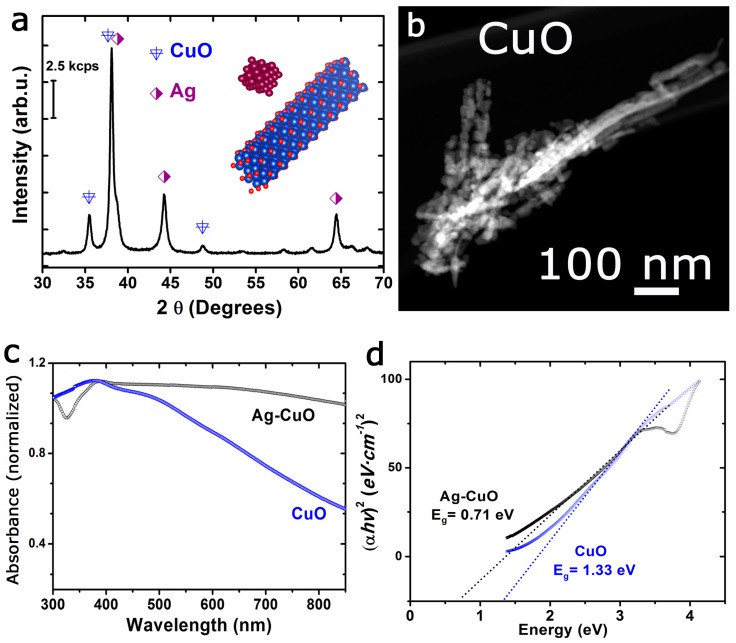
Additional characterization of the photocatalytic materials: (**a**) X-ray diffractogram of the Ag-CuO hybrid material accounting for the presence of both cubic and monoclinic crystallographic phases for silver and copper oxide, respectively; (**b**) HAADF-STEM representative image of the silver-free CuO nanostructures; (**c**) UV-Vis-Near Infrared absorption spectra of the Ag-CuO and CuO nanomaterials; (**d**) Tauc plots for the determination of the band gap energies for Ag-CuO and CuO structures assuming an indirect transition.

XRD analysis also corroborated the presence of both silver and copper oxide crystalline phases assigned to a cubic (Fm3m) and a monoclinic (C2/c) system, respectively (Figure 2a). The optical characterization of the Ag-CuO nanohybrids by UV-Vis-NIR spectroscopy revealed a broad absorption spectrum expanding towards the visible and near-infrared (NIR) range (Figure 2c). The silver-free CuO nanotubes synthesized as control (Figure 2b) exhibited similar optical absorption properties in the visible range, although it did not expand beyond the visible range and decayed in the NIR region (Figure 2c). The energy band gap for both the CuO and Ag-CuO nanostructures was determined from the optical absorption near the band edge using the classical Tauc approach and assuming an indirect band gap semiconductor system where α·E*_photon_* = *K* (E*_photon_*− E_g_)^1/2^ (being E*_photon_* and E_g_ the discrete photon energy and the band gap energy, respectively). The estimated band gap energy was calculated at 1.33 eV for the CuO nanostructures and 0.71 eV for the Ag-CuO hybrids (Figure 2d). These values were lower than the band gap reported for bulk CuO structures (typically 1.4 eV) and confirmed the potential optical response of these structures in the visible-NIR range [53].

### 3.2. Photocatalytic Performance of the Ag-CuO Heterostructures for N-Hexane Total Oxidation

**Figure 3:**
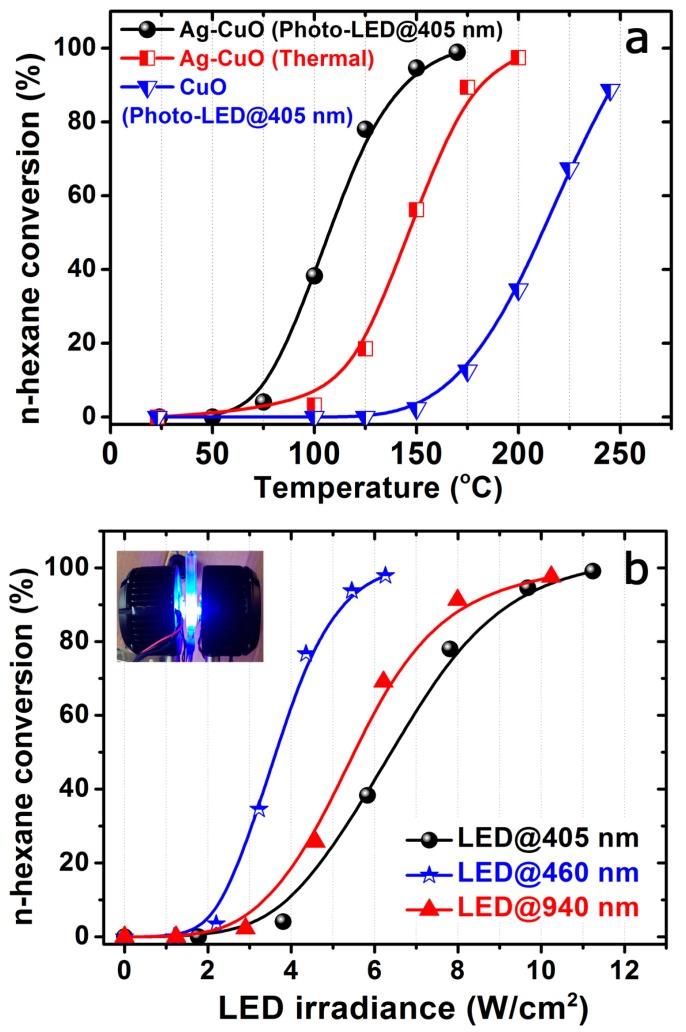
LED-driven photocatalytic oxidation of n-hexane: (**a**) n-hexane conversion curves obtained after photocatalytic activation of Ag-CuO (spherical symbols), CuO (triangle symbols) with a LED emitting at 405 nm, and alternatively after thermal heating of Ag-CuO with a conventional heating setup (square symbols); (**b**) n-hexane light-off curves under different irradiation wavelengths as a function of the irradiance (in W/cm^2^) specifically required for each LED; (inset: Digital image of the 405 nm LEDs simultaneously irradiating the quartz cuvette reactor).

Figure 3 shows the photocatalytic response of the Ag-CuO hybrid towards the oxidation of n-hexane under illumination with a high irradiance LED emitting at 405 nm (see inset in Figure 3b). Total oxidation was achieved at temperatures below 180 °C. Remarkably, light-off oxidation curves started at temperatures below 50 °C and T_50_ (Temperature of reaction required to reach 50% of conversion) remained below 100 °C. These results contrast with the photocatalytic behavior identified for the silver-free CuO counterpart under similar LED irradiation conditions. In this latter case, temperatures above 200 °C were necessary to reach match T_50_ and a complete n-hexane oxidation was not achieved (maximum 90% conversion, see Figure 3a). Finally, it is worth mentioning that the thermal catalytic experiment (in the absence of LED irradiation) with the Ag-CuO catalyst yielded higher n-hexane conversion levels than the CuO catalyst but were less effective than the photocatalytic experimental conditions (Figure 3a).

Additional photocatalytic experiments were carried out with the Ag-CuO heterostructure under illumination with different LED wavelengths at 460 and 940 nm, respectively. The use of LEDs emitting in the visible and NIR ranges rendered equivalent n-hexane photo-oxidation levels and analogous overlapping light-off curves to the one displayed in Figure 3a (data not shown). The major differences were observed in terms of the LED power density required in each experiment to achieve those conversion levels. Figure 3b summarizes the LED power irradiance (expressed in W/cm^2^) required at 405, 460, and 940 nm, respectively. Upon comparison of the three LEDs, it became clear that the photocatalytic efficiency was higher at 460 nm. The irradiation under the LED emitting at 405 nm required almost double irradiance to reach full photo-conversion of n-hexane. We observed a stable photo-response after multiple cumulative reaction runs performed under different LED wavelengths and no evidences of deactivation.

## 4. Discussion

The positive photocatalytic response towards n-hexane oxidation of the present Ag-CuO hybrid structures can be justified in terms of the synergetic combination of plasmonic silver and the visible-light response of the p-type semiconductor CuO. In contrast to previous Ag-Cu systems [55,108,109,111,114,117,119], our synthesis methodology enables the generation of a well entangled hybrid system where Ag domains of different sizes and morphologies are perfectly encapsulated within CuO nanotubes (Figure 1 and Figure 2). The most plausible mechanism for the formation of this hybrid is the thermal decomposition of an unstable silver-copper mixed oxide Ag_x_Cu_y_O_z_ that evolves into the corresponding silver and copper oxides counterparts [125,126,127]. The thermal treatment at high temperature during the preparation also favored the subsequent thermal decomposition of Ag_2_O into metallic Ag and oxygen at temperatures in the range of 195–205 °C and enabling partial mobility of silver throughout the CuO tubular scaffold (Figure 1) [76,128].

**Figure 4:**
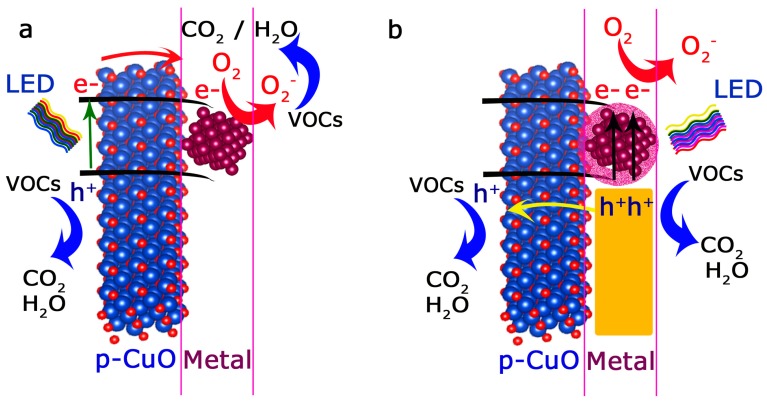
Schematic diagram illustrating the most plausible charge-transfer and photocatalytic mechanisms in the metal/p-type semiconductor Ag-CuO hybrids: (**a**) If the LED excitation wavelength is larger than the energy band gap of CuO, electrons from the valence band can be excited to the conduction of CuO and subsequently transferred and trapped by Ag energy levels; (**b**) Ag plasmon-induced charge transfer by hot holes injection into the p-type CuO energy levels.

To justify the full-range response under different LED wavelengths (Figure 3b), a combination of different photo-excitation and charge-transfer mechanisms can be taking place [68,69,81,99,129,130]. The different photocatalytic response observed after comparison between Ag-CuO and CuO irradiated with the 405 nm LED (Figure 3a) clearly demonstrates the positive influence of the silver entangled nanostructures. Given the heterogeneous disposition of silver entities, different photo-activation pathways can be simultaneously occurring in our catalysts. First of all, a fraction of smaller silver domains (Figure 1b–d) with the proper energy levels can be acting as sinks or trap centers for the electrons photogenerated by the CuO semiconductor fraction (Figure 4a). As a result, the expected high electron-hole recombination rates of CuO can be inhibited and/or partially delayed. Therefore, the unpaired holes remain available in the valence band of CuO to readily oxidize n-hexane molecules (Figure 4a). Likewise, electrons in the Ag surface can participate in the formation of reactive superoxide anions or relax via thermal energy dissipation [95,131]. The superoxide anions may subsequently react after their photo-induced dissociation (Figure 4a) and contribute to the oxidation of the n-hexane molecules [5,68,69,76,81,105,130,132].

Another fraction of silver structures with different sizes and anisotropic shapes (i.e., rod-like) that remain embedded within the CuO tubular matrix (Figure 1e,g,i) can provide additional plasmon-driven photo-excitation pathways. Metallic silver nanostructures are considered as excellent plasmonic materials [68,95,133]. The valence electron clouds present in their metal surfaces can oscillate and resonate at different frequencies in the UV-Vis-NIR ranges generating localized surface plasmons (LSPR). The surface plasmons can interact with the CuO nanotubes via radiative damping mechanisms that imply the reemission and/or trapping of light from the metal to the surrounding semiconductor matrix [95,133]. This approximation would be more likely to occur with the larger Ag domains isolated within the CuO nanotubes (Figure 1e–i) where scattering phenomena would be more plausible (Figure 2c) [95,131]. The photocatalytic response under 940 nm wavelengths can be also tentative attributed in part to the presence of rod-shaped Ag structures entangled within the CuO matrix (Figure 1g and Figure 3b). These anisotropic silver nanostructures exhibit plasmon absorbance at longer wavelengths than spheres or cubes (Figure 1g) [68]. The presence of silver expands the absorption and light-trapping capabilities in the visible-NIR range, thereby expanding the potential exploitation of the solar energy (more than 80% of the solar spectrum range) [68,92].

Surface plasmons can alternatively decay via non-radiative pathways involving the generation of electron-hole pairs by interband and/or intraband excitations [68,81,92,95]. In this scenario, Ag and CuO entangled interfaces can form a metal/p-type semiconductor Schottky barrier for holes after matching their Fermi levels [3,55,119]. The excitation with sufficiently high energy LEDs (i.e., 405 and 460 nm, Figure 3 and Figure 4b) enables the plasmon-induced injection of hot holes from the silver bands into the valence band of the CuO p-type semiconductor (Figure 4b). These high energetic holes are able to pass the Schottky barrier and rapidly react with the n-hexane. The reduced dimensions of the heteronanostructures minimize the probability of undergoing another relaxation/recombination process [68]. While the injection of hot electrons (using n-type semiconductors such as TiO_2_) is the most accepted plasmon-driven charge transfer mechanism, there exist recent interesting studies claiming the importance of hot holes in other plasmonic-based systems [73,86,92,97,134,135,136,137] such as Au-NiO_x_, Au-pGaN [73], Au nanorods coated with a CoO nanoshell [138], Au nanostructures [101,139,140], or Ag-BiOCl hybrids [86,137].

We tentatively propose a combination of the different photocatalytic mechanisms given the diversity of Ag domains. Indeed, the better photoresponse of the Ag-CuO hybrid in comparison with the CuO nanostructures confirms the important role of silver as plasmonic structure to harvest light in the whole visible to NIR range. Likewise, the close contact between both metal and semiconductor phases has enabled a suitable interfacial contact to promote electron and holes mobilities and minimize undesired recombination and relaxation pathways. In summary, we can conclude that our Ag-CuO represents a very attractive metal/p-type semiconductor candidate with full-spectrum response that can be envisioned as an affordable alternative for green and sustainable photo-assisted chemistry, with special attention to energy and remediation processes.
